# Dosimetric effect of six degrees of freedom couch top with rotational setup error corrections in proton therapy

**DOI:** 10.1002/acm2.14043

**Published:** 2023-05-30

**Authors:** Takahiro Kato, Sho Sasaki, Tomohiro Ikeda, Ryohei Kato, Masato Kato, Yuki Narita, Sho Oyama, Shinya Komori, Takaomi Harada, Masao Murakami

**Affiliations:** ^1^ Department of Radiological Sciences School of Health Sciences Fukushima Medical University Fukushima Japan; ^2^ Department of Radiation Physics and Technology Southern Tohoku Proton Therapy Center Fukushima Japan; ^3^ Department of Radiation Oncology Southern Tohoku Proton Therapy Center Fukushima Japan

**Keywords:** proton beam range, proton therapy, six degrees of freedom couch

## Abstract

**Purpose:**

To investigate the dosimetric effect of six degrees of freedom (6DoF) couch top with rotational corrections in proton therapy (PT).

**Methods:**

The water equivalent thickness (WET) was measured using a proton beam with a 6DoF couch top and patient immobilization base plate (PIBP) placed in front of a motorized water phantom. The accuracy verification was performed with the beam axis set perpendicular to the 6DoF couch top and tilted in 10° steps from 10° to 30°. Up to 3° rotational correction may be added during the actual treatment to correct the rotational setup error on our system. The measured and calculated values using the treatment planning system were compared. Additionally, the effect of the 3° difference was evaluated using actual measurements concerning each angle on the proton beam range.

**Results:**

The WET of the 6DoF couch top and PIBP were 8.5 ± 0.1 mm and 6.8 ± 0.1 mm, respectively. The calculation and the actual measurement at each angle agreed within 0.2 mm at the maximum. A maximum difference of approximately 0.6 mm was confirmed when tilted at 3° following 30° with the 6DoF couch top plus PIBP.

**Conclusions:**

The dosimetric effect of the 6DoF couch top with rotational corrections in PT differs depending on the incidence angle on the couch top, and it increased with the increased oblique angle of incidence. However, the effect on the range was as small as 0.6 mm at the maximum. The amount of rotational correction, the angle of incidence of the beam, and the effect of rotational corrections on the proton beam range may differ depending on the structure of the couch top. Therefore, sufficient prior confirmation, and subsequent periodical quality assurance management are important.

## 1 INTRODUCTION

Proton beams carry charged particles that deposit relatively low doses in the entrance path proximal to the tumor and deposit most of their energy at the end of its path, called the Bragg peak. Generally, proton beams reduce the integral dose, compared with photons. A rotating gantry that can irradiate from all directions and an image‐guided technology that ensures position accuracy are indispensable to effectively utilize the excellent proton beam physical characteristics. Six degrees of freedom (6DoF) couch is essential for accurate patient positioning,^1^, ^2^ and the required specification is to get geometrical errors as close to zero as possible by remotely repeating fine adjustments.

The patient is laid on the couch in the supine position, and irradiation is performed by selecting the optimum angle using a rotating gantry. Patient immobilization devices and treatment couch top on the beamline should always be considered in external radiation therapy; however, special attention is necessary for proton therapy (PT) due to the finite range of proton beams.^3^, ^4^, ^5^, ^6^ The couch top crosses the beamline, especially when selecting the gantry angle from the posterior direction during the supine position on the couch; therefore, effect on the range should be considered. These are usually reflected in the calculation by including them in the contour or by virtually inserting them as the couch top on the treatment planning system (TPS).^7^, ^8^, ^9^, ^10^ In general, the function of the virtual couch on the TPS can accurately estimate the effect on the range by adjusting the Hounsfield unit values. However, rotation is added to the couch top during actual treatment to correct the patient rotational setup error, which can be a factor of uncertainty because it cannot be reproduced by the virtual couch on the TPS. The degree of uncertainty depends on the geometry, material, and amount of rotation of the couch top; however, a detailed report on this issue has not been elucidated thus far. Therefore, we investigated the dosimetric effect of the 6DoF couch top with rotational setup error corrections in PT.

## 2 MATERIALS AND METHODS

### 2.1 Basic property of our treatment couch

**FIGURE 1:**
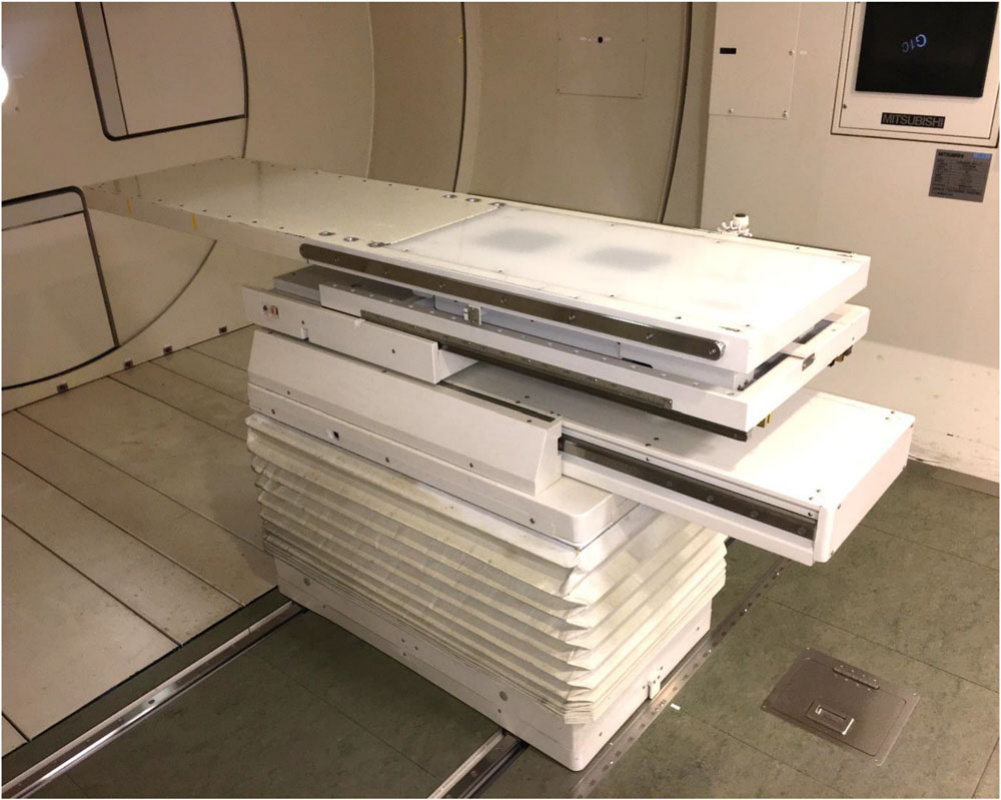
An overview of the six degrees of freedom couch.

This study used Hitachi's proton‐type Particle Therapy System (Hitachi, Kashiwa, Japan) which comprised an ion source, 3‐MeV radiofrequency quadrupole linear accelerator, 235‐MeV synchrotron, high‐energy beam transport line, two gantry irradiation rooms, and one irradiation room for the horizontal beam. Beam‐wobbling magnets, lead scatterer, main dose monitor, ridge filter, range shifter, backup monitor, flatness monitor, block collimator, multi‐leaf collimator, and range compensator comprised the proton beam delivery system. Two dipole magnets comprised the wobbler system, and scattering elements create a broad, flattened beam at the final aperture. This method is known as the single‐ring wobbling method, which is one of the passive scattering methods.^11^, ^12^ The treatment couch has a 6DoF structure, which is not a robotic couch. The overview of the treatment couch is shown in Figure 1.

**FIGURE 2:**
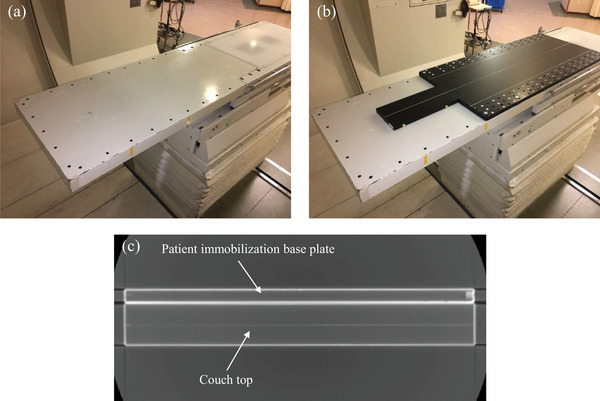
Overview of the (a) couch top and (b) patient immobilization base plate (PIBP) on the couch top (couch top plus PIBP). (c) Axial image of computed tomography of the couch top plus PIBP.

A total of three types of couch tops can be selected for this couch, depending on the purpose, including one for the head and neck and two for the trunk. The standard type shown in Figure 2a is used when the patient lies on the couch in a supine position and uses the anterior beam only to the head and neck, or when using a beam other than the posterior oblique to the trunk. This study analyzed this standard type, which is most frequently used in actual treatment. This couch top is Hitachi's original specification. Here, this standard type is referred to as a couch top, and the plate for fixing the thermoplastic shell is referred to as a patient immobilization base plate (PIBP) (Engineering System, Nagano, Japan). The couch top is 500 × 960 × 60 mm in size, covered with carbon‐fiber‐reinforced plastics (CFRP) with a 1‐mm thickness, and the inside is filled with acrylic foam. A PIBP for fixing the thermoplastic shell is fixed on the couch top and used when a thermoplastic shell is used. This is shown in Figure 2b, with a 20‐mm thickness, covered with CFRP with a 1.5‐mm thickness, and the inside is filled with acrylic foam.

### 2.2 Verification of couch modeling accuracy

**FIGURE 3:**
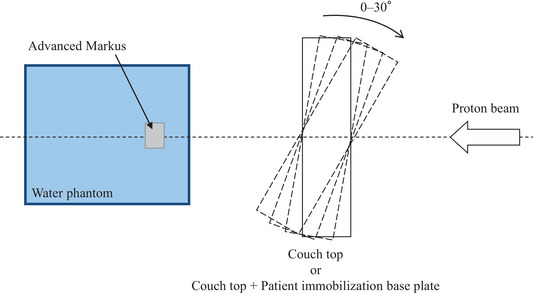
Schematic diagram of the experimental setup for water equivalent thickness measurements of the six degrees of freedom couch in 10° steps from 10° to 30° perpendicular to the couch top.

A commercially available TPS, XiO‐M (Hitachi, Kashiwa, Japan), was used to calculate the PT dose distributions. PT plans were calculated using a pencil beam algorithm with a calculation grid spacing of 1 mm. The effect of the beam selected through the couch top or couch top plus PIBP must be considered in the treatment planning. The couch top and PIBP in the beam path act as range shifters for PT.^5^ Therefore, it was registered in the TPS as a virtual couch function. Virtual couch modeling is performed by adjusting Hounsfield unit values of the couch top and PIBP. Measurement was performed using a proton beam because information on the water equivalent thickness (WET) of the couch top or PIBP is required to validate virtual couch modeling. The ranges of the 150‐ and 210‐MeV wobbled proton beams were measured with and without the couch top or PIBP installed to be completely perpendicular to the beam axis. The field size was set to 10 × 10 cm on the isocenter plane, and the measurement was performed under the condition that the isocenter was aligned with the phantom entrance surface. The WET was derived from the difference in the range measured with and without the couch top or PIBP. Here, the definition of the range is the depth along the beam central axis in water to the distal 90% point of the maximum dose value. The same definition was used for both actual measurement and calculation. The posterior direction beam passes through the couch top or couch top plus PIBP when used: however, measuring the range of the proton beam in this state is technically difficult. Therefore, the WET was measured using a proton beam with couch top or PIBP placed in front of a horizontally driven customized one‐dimensional water phantom (TOYO MEDIC Co., Ltd. Tokyo, Japan) and Advanced Markus chamber (Type 34045) (PTW, Freiburg, Germany) with a gantry angle of 90°. This system can measure depth‐dose profiles in water with a resolution of 0.1 mm. The reproducibility determined through multiple measurements of the same location was confirmed to be better than ± 0.02 mm from preliminary experiment results. A completely horizontal stand was installed on the floor, and the couch top, or PIBP was placed on it with high precision using lead blocks as supports. The measurement points were sampling six different locations along the couch top or PIBP. The average value of the obtained results was referenced during couch modeling in the TPS. Moreover, computed tomography (CT) was employed for a visual evaluation of the uniformity of the internal structure of the entire couch top and PIBP.^4^ Figure 2c shows CT transverse cross‐sections of the couch top plus PIBP. The calculated and measured values were compared and examined to evaluate the accuracy after registering in the TPS. The accuracy verification was performed with the beam axis set perpendicular to the couch top assuming actual clinical use and tilted in 10° steps from 10° to 30°. The basic experimental setup is shown in Figure 3. This figure shows a top view. Confirmation was performed under a total of eight conditions because performing with only the couch top and the PIBP attached to the couch top is necessary. Each measurement was performed three times on different days, and the average value was calculated. The modeling accuracy of the couch and the rotational setup error correction are among the factors that influence the uncertainty of the proton beam range. Therefore, we examined this point in the next section.

### 2.3 Effect of rotational setup error correction

**FIGURE 4:**
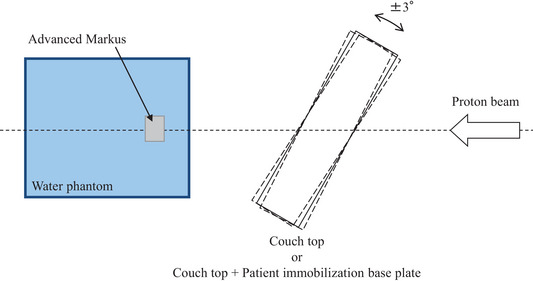
An example schematic diagram of the experimental setup for water equivalent thickness measurements in 30° ± 3° beam incident angle.

A virtual couch is set and the calculation is performed in the treatment plan when the beam passes through the couch top or couch top plus PIBP. Up to 3° rotational setup error correction may be added during the actual treatment to correct the rotational setup error on our system. Thus, a geometrical difference of up to 3° may exist from the state set on the TPS. The effect of the difference of 3° on the range was evaluated by actual measurement. The evaluation method measures the WET with tilted ± 3° for each of the same four conditions as in the previous section (Figure 4) and determines the amount of change of WET from the state with no tilting. A sheet with an enlarged copy of the protractor was set on the horizontal stand, and the couch top, and PIBP were accurately set using that as an index. The accuracy of the protractor sheet was confirmed using a digital goniometer TL792‐JP (Neoteck, CA, USA) with a resolution of 0.05°. Each measurement was performed three times on different days, and the average value was calculated.

## 3 RESULTS

**FIGURE 5:**
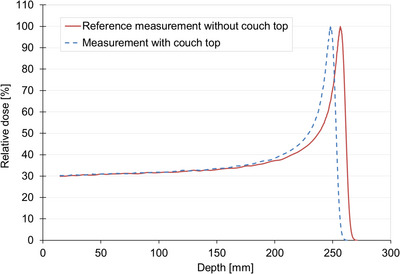
Depth‐dose curves with and without the couch top. The water equivalent thickness of the couch top from the range shift of 210‐MeV wobbled proton beam is approximately 8.5 mm.

**FIGURE 6:**
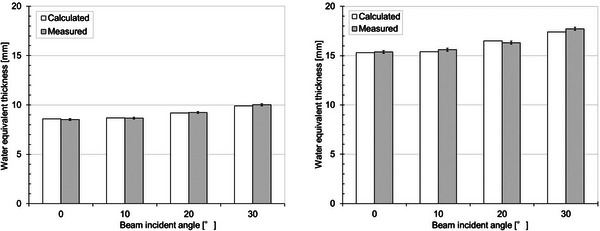
Comparison of calculated and measured water equivalent thickness of the couch top only (left panel) and couch top plus patient immobilization base plate (right panel) in each beam incident angle. The error bar indicates the ± 1σ.

**FIGURE 7:**
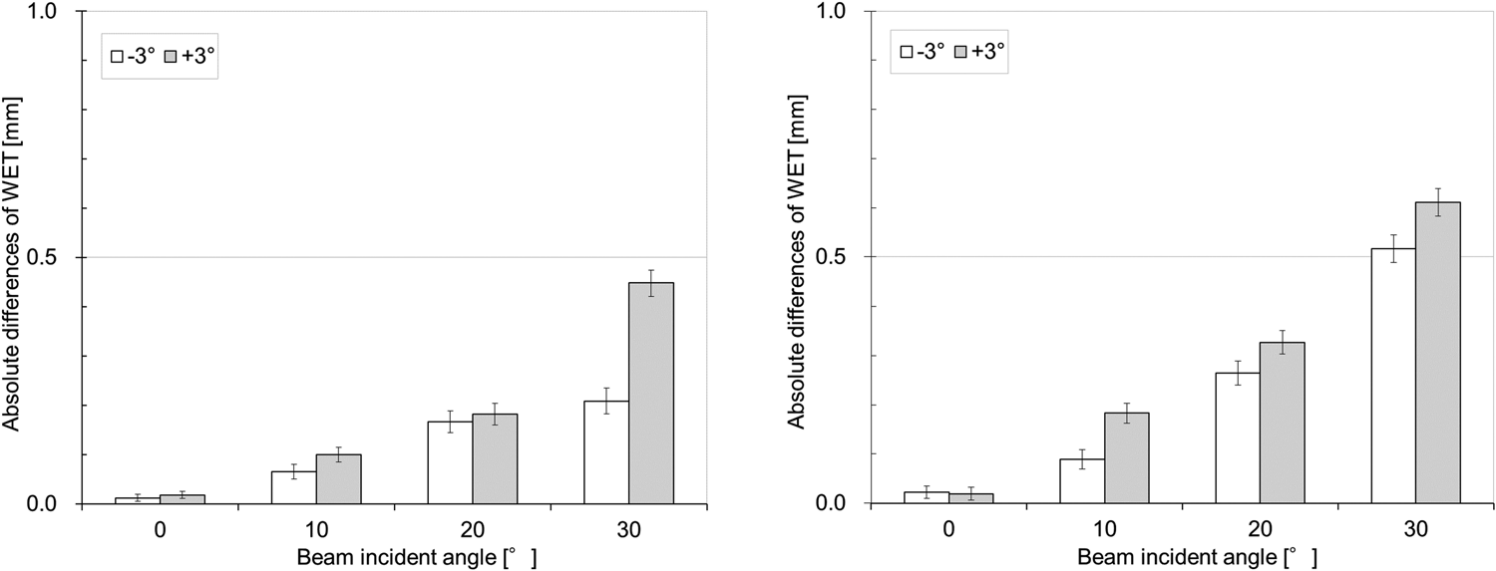
Absolute differences in water equivalent thickness (WET) of the couch top only (left panel) and couch top plus patient immobilization base plate (right panel) when tilted at ± 3° for each incident angle. The error bar indicates the ± 1σ.

Figure 5 shows an image of the typical results of the depth‐dose profile of the 210‐MeV wobbled proton beam measured with and without the couch top. The measured WET of the couch top and PIBP were 8.5 ± 0.1 mm and 6.8 ± 0.1 mm, respectively. The difference between the mean values of the WET at six different locations was <0.2 mm at the maximum. The measured WET obtained using 150‐ and 210‐MeV wobbled proton beams were confirmed to be in good agreement. The whole image was evaluated using CT and was visually confirmed, but with no particular problem. The calculation and actual measurement results for each angle are shown in Figure 6. The calculated WET of the couch top and PIBP at beam incident angle 0° was 8.6 mm and 6.7 mm, respectively, after careful couch modeling in the TPS. The calculation and the actual measurement at each angle were in agreement with 0.2 mm at the maximum. Figure 7 shows the result of the maximum value of the change when tilted ± 3° concerning each angle and when not tilted. Differences are shown here as absolute values. A maximum difference of approximately 0.6 mm was confirmed when tilted at 30° with the couch top plus PIBP.

## 4 DISCUSSION

The use of a rotating gantry that can irradiate from 360° is standard in photon therapy and PT. The effect of the couch present before it enters the patient cannot be ignored; thus, it is generally calculated by setting a virtual couch on the TPS.^5^ Some studies report on virtual couch modeling and its accuracy in photon beam,^7^, ^8^, ^9^ but rather limited in PT.^10^ However, modeling accuracy is more important for proton beams with ranges. The couch capable of 6DoF correction has become widespread to correct setup errors more accurately, in recent years. Having a rotating system correction function as well as translation to correct patient setup errors is necessary. However, the addition of rotational setup error correction may cause a difference in the beam path from the treatment planning, which may affect the dose distribution. To our best knowledge, this study is the first to report detailed analysis of the dosimetric effect of 6DoF couch top with rotational setup error corrections in PT.

Our system has up to 3° and 2° corrections for rolling and pitching, respectively. Therefore, the change in range when adding an angle of 3° was evaluated, which is expected to have the greatest effect. The results confirmed that the maximum effect was approximately 0.6 mm under the conditions verified in this study. The relatively small effect was due to the internal structure of the couch top and PIBP. The couch top is relatively thick with a 60‐mm thickness to provide rigidity; however, the inside has a urethane foam structure, and the Hounsfield unit value is approximately −970 HU, which is close to air. The PT system has not yet been standardized, and the structure of the couch differs from system to system. The manufacturing of homogeneous and low‐density table tops is becoming the industry standard with the development of foam core technology and carbon fiber overlay.^3^ However, McCormack et al. emphasized that the structure of the couch insert might not be perfectly uniform.^13^ Therefore, sufficient preliminary verification is considered essential because of the variations in the manufacturing process.

The posterior beam incidence angle is limited to ± 30° from the angle perpendicular to the couch surface when using the standard couch top at our institution. The snout position will be extremely far away and the dose distribution will greatly deteriorate, or the edge of the couch will enter the beamline and the correction will malfunction if it exceeds this range. The range of the angle of beam incidence concerning the couch may vary from system to system. Hence, the larger the angle, the greater the effect of the correction.

This study confirmed the increased uncertainty of the range when the beam is incident through the couch top or couch top plus PIBP. Our system had a maximum correction angle of 3°, but other systems may have higher angles. The larger the correction angle, the greater the effect. The structure of the couch and the incident angle of the beam, prior verification, and regular quality assurance (QA) are required especially for proton beams with a range since the degree of influence varies depending on the maximum correction angle of the system used. Thereafter, it should be reflected in the distal and proximal margins for treatment planning. Additionally, the initial patient setup should be carefully performed to prevent extreme setup errors during patient setup.^2^

This study has limitations. Only the effect of the correction for each axis can be considered. Notably, in reality, rolling, and pitching corrections occur in combination in addition to the loaded state; hence, the effect may be even greater in worst‐case scenarios. Recognizing and responding to the uncertainty in the range of the proton beam is important when setting the proton beam through the couch. Moreover, periodical QA is necessary as reported in this study.

## 5 CONCLUSION

The dosimetric effect of the 6DoF couch top with rotational setup error corrections in PT was verified by actual measurement. The effect of rotational setup error correction on the proton beam range differs depending on the angle of incidence on the couch top and PIBP, and it increased with the increased oblique angle of incidence; however, the effect on the range was 0.6 mm at the maximum. The amount of rotational setup error correction, the angle of incidence of the beam, and the effect of rotational setup error correction on the proton beam range may differ depending on the structure of the couch top; thus, sufficient prior confirmation, and subsequent periodical QA management are important.

## AUTHOR CONTRIBUTIONS

Takahiro Kato: Conceptualization, Investigation, Methodology, Writing–Original Draft. Sho Sasaki: Resources. Tomohiro Ikeda: Methodology. Ryohei Kato: Methodology. Masato Kato: Methodology. Yuki Narita: Writing–Review & Editing. Sho Oyama: Validation. Shinya Komori: Resources. Takaomi Harada: Formal analysis. Masao Murakami: Writing–Review & Editing.

## CONFLICT OF INTEREST STATEMENT

The authors have no relevant conflicts of interest to disclose.
